# Ledderhose Disease: Clinical, Radiological (Ultrasound and MRI), and Anatomopathological Findings

**DOI:** 10.1155/2015/741461

**Published:** 2015-09-06

**Authors:** Y. Omor, B. Dhaene, S. Grijseels, S. Alard

**Affiliations:** Service d'Imagerie Médicale, CHU Saint-Pierre, 1000 Bruxelles, Belgium

## Abstract

Plantar fibromatosis, or Ledderhose disease, is a rare hyperproliferative disorder of the plantar aponeurosis. It may occur at any age, with the greatest prevalence at middle age and beyond. This disorder is more common in men than woman and it is sometimes associated with other forms of fibromatosis. Diagnosis is based on clinical examination. Ultrasound (US) and magnetic resonance imaging (MRI) can be useful to confirm the diagnosis. A 44-year-old man with Ledderhose disease who underwent ultrasound and MR is described in this paper.

## 1. Introduction

Plantar fibromatosis, or Ledderhose disease, is a fibrous proliferation arising within the plantar fascia end exhibits typical clinical nodular features [1, 2]. This is a benign disease, but difficult to treat. The lesion appears as a firm single thickening or a nodule, occasionally painful, localized to the medial portion of the sole of the foot. This unusual condition has not been extensively studied with US and MRI, and very little has been written about it. In this paper, we report on the imaging of Ledderhose disease (ultrasound and MRI) [1].

## 2. Case Report

A 44-year-old man with no significant medical history presented with an 8-year history of painless plantar nodule of the right foot. In the previous 6 months the patient felt discomfort while walking. On examination, he had subcutaneous nodules on the medial aspect of the plantar surface of the right foot. There was no family history of similar nodules. The patient was not taking any medication and was otherwise healthy. Vital signs and laboratory tests were normal.

**Figure 1:**
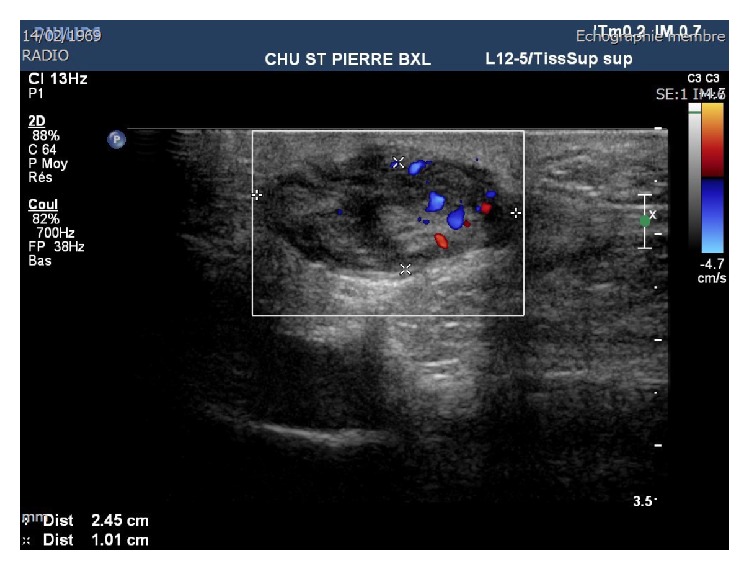
An ultrasound of the plantar arch was made and found a hypoechoic and homogeneous nodule at the thickness of the plantar fascia with a significant hyperemia in Doppler.

**Figure 2:**
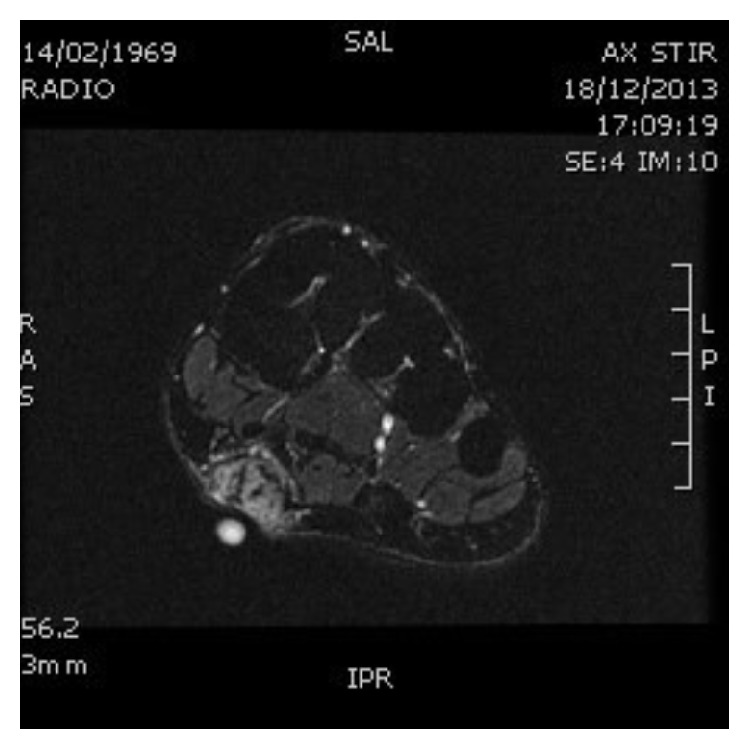
MRI of the right feet showing a nodule hyperintense in weight after saturation proton density fat signal.

**Figure 3:**
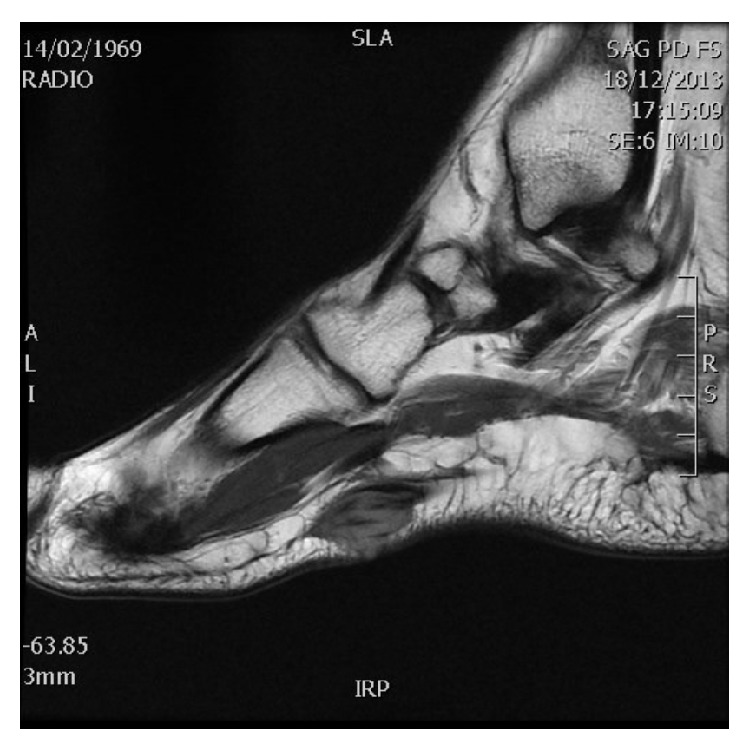
MRI of the right feet: sagittal T1-weighted image demonstrated a nodular soft-tissue masse in the distal plantar fascia, which are hypointense to muscle.

**Figure 4:**
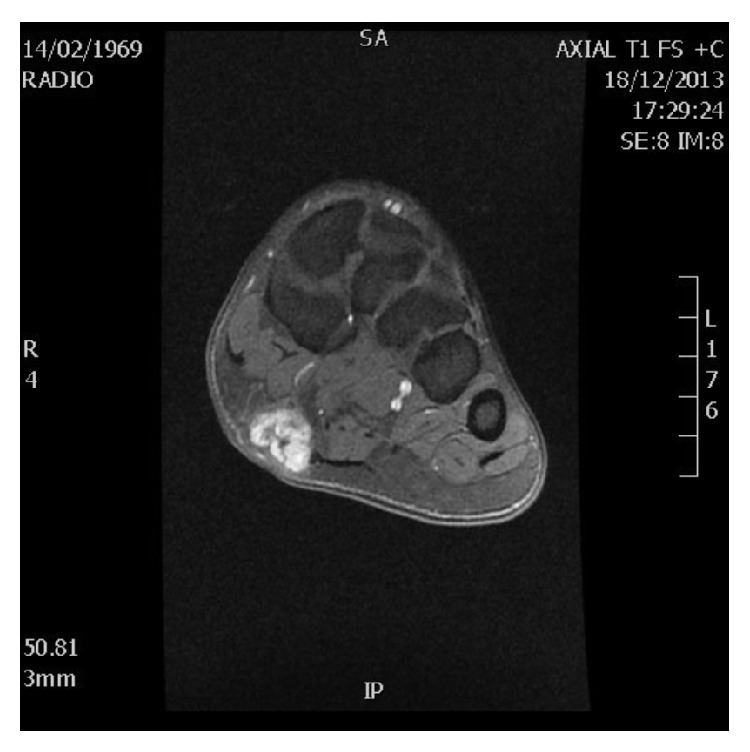
MRI of the right feet: axial T1-weighted image showing nodular soft-tissue masse sitting in the thickness of the distal part of the superficial medial plantar fascia and enhancing intensely Gadolinium.

An ultrasound (Figure 1) of the plantar arch was made using 13–5 MHz array transducer and showed a hypoechoic and homogeneous nodule of the plantar fascia with increased color Doppler flow. MRI of the foot confirmed the ultrasound data, showing a hyperintense nodule on proton density images (Figure 2) which was hypointense on T1-weighted images (Figure 3), at the distal part of the superficial medial plantar fascia. The lesion was intensely enhanced after injection of Gadolinium (Figure 4).

**Figure 5:**
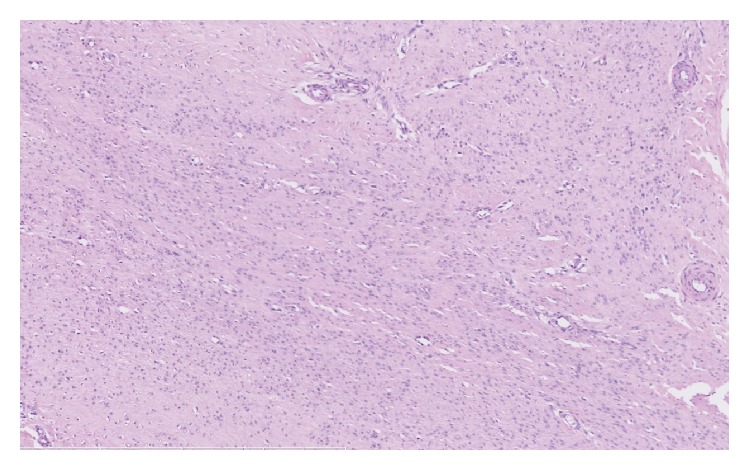
Microscopic view showing of a nodule demonstrated increased fibroblastic activity and a reduction of the collagen network (H&E, magnification ×10).

The diagnosis of Ledderhose disease was suspected based on the clinical-radiographic data. However, because of the flow inside the lesion at color Doppler, a biopsy was performed to rule out malignancies. Histological examination (Figure 5) of the nodule demonstrated increased fibroblastic activity and a reduction of the collagen network. Our patient received conservative treatment with anti-inflammatory drugs, physiotherapy, and orthotic support with satisfactory evolution.

## 3. Discussion

Ledderhose disease is a rare, benign, hyperproliferative disorder of the plantar aponeurosis [2, 3]. It was initially described by the German physician Georg Ledderhose in 1897 [4]. First ultrasonographic description was made by Reed in 1991 [5].

The etiopathology of Ledderhose disease is still uncertain [2]. A genetic predisposition and alteration in the collagen profile of the plantar fascia have been proposed as causative factors [5]. It is likely that the increased production of some cell growth factors may affect the formation of fibromatosis and can cause the progressive worsening of the contracture, as in Dupuytren's disease [2].

Caucasians are more affected often than any other ethnic group starting from the sixth decade. It is well recognized in adults and is very rare in children [2]. Men are affected twice as often as women. Only 25% of patients show bilateral involvement [2, 4]. Ledderhose disease is often associated with diseases such as frozen shoulder, Dupuytren's disease, alcohol addiction, epilepsy, diabetes mellitus, and penile fibromatosis [4].

Ledderhose disease is characterized by slow-growing nodes in the central medial plantar fascia, which can lead to shrinkage and sclerosis of the entire plantar fascia. Not typically, but in rare cases, the fibromatosis leads to toe contractures. Symptoms include pain and swelling in the foot, which can lead to walking disability. The first signs of the early stage are local pressure and distension. The late stage is characterized by the formation of nodules and contractures of the plantar fascia [4].

The diagnosis of plantar fibromatosis is usually a clinical one and rarely requires confirmation [6].

Plantar fibromatosis exhibits almost a pathognomonic pattern, and US proved to be a quick, noninvasive, and cost effective technique to confirm the clinical diagnosis. The nodule is typically single and isoechoic, with a maximum diameter of about 1 cm, showing a heterogeneous internal structure with a few thin hyperechoic septa. The nodular fibrous proliferation adheres to the major axis of the plantar fascia; and it exhibits clear-cut margins. No calcifications or fluid collections are seen within the nodule. Color and power Doppler usually show no flow inside [4].

On MR imaging, plantar fibromatosis appears as a well-defined nodule that is contiguous with the plantar fascia and has low signal intensity on T1-weighted sequences and low-to-intermediate signal intensity on T2-weighted sequences. MR imaging is excellent at showing the deep extension found in advanced, aggressive forms of plantar fibromatosis, but the availability and low cost of sonography make it the imaging technique of choice for most patients [7].

Thus MRI has an important role in both the diagnosis and assessment of the severity of the lesions, thereby guiding appropriate clinical management [8].

Differential diagnosis of a mass on the sole of the foot includes plantar fasciitis (the most frequent lesion of a swelling of the plantar fascia), leiomyoma, rhabdomyosarcoma, neurofibroma, and liposarcoma. Correlation of clinical, radiological, and histological findings helps in confirming a diagnosis [8].

Clinical and pathologic studies have classified plantar fibromatosis into three stages: proliferative, involutional, and residual. The first stage is described by cellular proliferation, the second stage by nodule formation, and the third stage by tissue contraction. Histologic findings associated with fibromatosis include myofibroblastic proliferation with elongated oval-shaped nuclei and a preponderance of type III collagen. Atypical mitotic figures, different shape of nuclei, and ill-defined bundles of spindle shaped fibroblasts suggest a higher degree of differentiation, helping to rule out fibrosarcoma. A histochemical, immunohistochemical, and ultrastructural study performed by Zgonis showed similar findings to those of Dupuytren's disease [6].

Conservative treatment includes padding, orthotic support, physiotherapy, collagenase treatment, and anti-inflammatory drugs, as well as intralesional steroid injections. The surgical options are chosen when conservative measures are ineffective and when the pain persists. The treatment could include local excision, wide excision, and complete plantar fasciectomy [2]. A wide excisional approach or subtotal plantar fasciectomy seems to offer the best prevention of lesion recurrence [6]. Recently, the importance of radiation therapy for primary treatment of Ledderhose disease has been investigated in a few studies [2].

## 4. Conclusion

Plantar fibromatosis is a benign lesion of unknown origin. The diagnosis of this disease is based on clinical examination. Radiographs are not necessary to establish the diagnosis, but the exclusion of bone disease may be indicated. Sonography and magnetic resonance imaging (MRI) can be useful to confirm the diagnosis. Biopsies may be performed to rule out malignancies.
